# A facile and efficient synthesis of 1,8-dioxodecahydroacridines derivatives catalyzed by cobalt–alanine metal complex under aqueous ethanol media

**DOI:** 10.1186/s13065-019-0545-3

**Published:** 2019-03-28

**Authors:** M. Mujahid Alam, Ahmed T. Mubarak, Mohammed A. Assiri, S. Merajuddin Ahmed, Ahmed M. Fouda

**Affiliations:** Department of Chemistry, King Khalid University, P. O. Box 9004, Abha, 61413 Saudi Arabia

**Keywords:** Cobalt–alanine metal complex, Multi component reaction (MCR), Dimedone, Ammonium acetate, 1,8-Dioxodecahydroacridine, One-pot synthesis

## Abstract

A facile and convenient method for the synthesis of acridines and its derivatives was developed through one-pot, three-component condensation reaction of aromatic aldehydes, 5,5-dimethyl-1,3-cyclohexanedione, aryl amines or ammonium acetates in the presence of a catalytic amount of cobalt–alanine metal complex using aqueous ethanol as a reaction medium is reported. The present described novel methodology offers several advantages over the traditional methods reported in the literature, such as mild reaction conditions, inexpensive catalyst, short reaction times, excellent yields of products, simplicity and easy workup are the advantages of this procedure.

## Introduction

Multi-component reactions (MCRs) have been paid much attention by synthetic organic chemists worldwide due to the effectiveness of multiple component reactions at building functionalized, novel drug discovery procedures and allow the fast, automated and high-throughput generation of organic compounds [1]. Thus we believe that discovering and developing new and novel bond formation of C–N, C–O and C–S bonds by MCRs is usual in numerous heterocyclic compounds is an important pursuit in pharmaceutical, biological and material science [2]. Consequently, the development of new multicomponent reactions towards biomedical and industrial scaffolds is inevitable at the present time. Therefore, in the last decade research in academia and industry has increasingly emphasized on the use of MCRs [3–11].

Acridine-1,8-dione and acridine derivatives are well known polyfunctionalized 1,4-dihydropyridines (DHPs) [12]. These derivatives of DHPs have an important ring skeleton and reported wide range of pharmaceutical and biological properties, including antitumor [13], antitubercular [14], antimalaria [15], antibacterial [16], antihypertensive [17], fungicidal [18], anticancer [19], anti-inflammatory [20] and diabetes [21]. These derivatives are also commercially used as calcium channel blockers [22–24]. Further, these compounds are also useful for the treatment of angina pectoris [25], hypertension [26–28] and Alzheimer’ disease [29]. Few of these compounds are used as effective drug for the treatment of congestive heart failure [30]. 1,4-Dihydropyridine derivatives are used in numerous bioactive compounds [31–33]. 1,8-Dioxo-decahydroacridines derivatives are using as dyes [34–36] and also as photoinitiators [37]. Furthermore, these derivatives have the applications in material science like semiconductors [38] and in spectroscopy as luminescent agent [39].

Due to the broad utility of acridines, there has been intense demand for the development of new and efficient synthetic protocols for the preparation of these important class of molecules have attracted a large number of organic chemists. Recently, many methods have reported in the literature for the synthesis of acridine derivatives containing 1,4-dihydropyridines, involves the three-component cyclocondensation reaction of 5,5-dimethyl-1,3-cyclohexanedione (dimedone), aromatic aldehydes and various aniline or ammonium acetate in the presence of a several catalysts such as Amberlyst-15 [40], sulfonic acid functionalized silica (SBSSA) [41], ammonium chloride, Proline [12], Zn(OAc)_2_·H_2_O or l-proline [42], triethylbenzylammoniumchloride(TEBAC) [43], ZnO nanoparticles [44], nano-Fe_3_O_4_ [45], CeCl_3_·7H_2_O [46], silica-bonded *N*-propyl sulfamic acid (SBNPSA) [47]. ionic liquids [48, 49] such as 1-methyl imidazolium trifluoroacetate ([Hmim]TFA) [50] and Bronsted acidic imidazolium salts containing perfluoroalkyl tails [51], microwave irradiation [52, 53], *p*-dodecylbenzenesulfonicacid (DBSA) [54] and PMA-SiO_2_ [55].

However, some of these reported methods for the synthesis of 1,8-dioxodecahydroacridine have limitations such as low yields, unpleasant experimental procedure, reagents are expensive or the use of an excess of catalyst, generation of polluting effluents and prolonged reaction times. Therefore, there is scope for further innovation of methods with milder reaction conditions, short reaction times, increase in variation of the substituents in the components and better yields for the synthesis of 1,8-dioxodecahydroacridine, the discovery of new methodologies using new and efficient catalyst is highly desirable.

In continuation to our effort in developing novel transition metal complexes with amino acids for various organic transformations [56–58], herein we would like to report an efficient method for the synthesis of 1,8-dioxodecahydroacridine derivatives through one-pot three component cyclisation reaction catalyzed by cobalt–alanine complex under an aqueous ethanol solvent system.

## Results and discussions

**Scheme 1:**
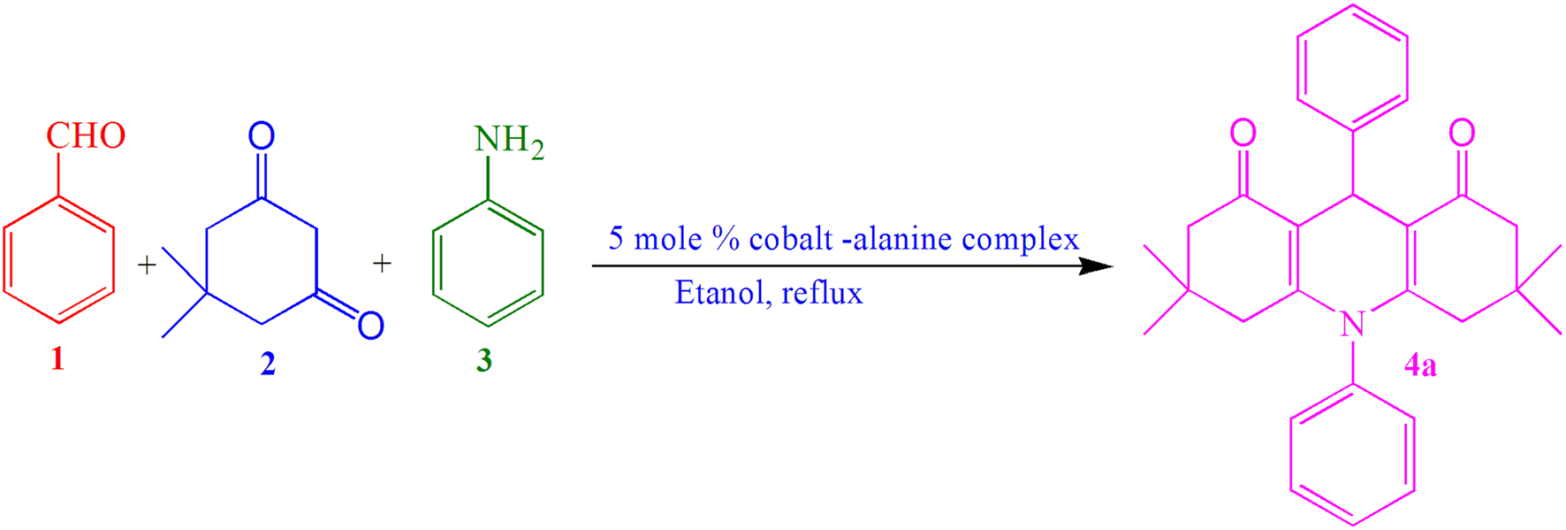
Synthesis of 1,8-dioxodecahydroacridines **4a** catalyzed by cobalt–alanine complex

Initially, we studied the one-pot three component condensation reaction of benzaldehyde (1 mmol), dimedone (2 mmol) and aniline (1 mmol) as a model reaction in the presence of cobalt–alanine complex (5 mol%) as a catalyst in aqueous ethanol solvent system and the product was obtained in excellent yield (Scheme 1).

After successful of the model reaction, we studied for the optimization of the reaction conditions with respect to temperature, time, solvent and molar ratio of the catalyst. It was observed that 5 mol% of catalyst was enough to complete the reaction and the desired 1,8-dioxodecahydroacridines products were obtained in high yield. The results are summarized in Table 1.

**Table 1:** Screening the amount of catalyst for the preparation of 1,8-dioxodecahydroacridines

Entry	Catalyst (mol%)	Time (h)	Yield (%)^a^
1	2.5	4.5	72
2	5	1.0	96
3	10	3.0	90
4	15	3.5	80

^a^Yields of isolated products

Further, we performed a screening of various solvents such as acetonitrile, DCM, DMF, EtOH and without solvent system for this reaction (Table 2, entries 1–5). we found ethanol (Table 2, entry 4) was the best solvent to afford the desired products in higher yield in shorter reaction time.

**Table 2:** Study of solvent system for the preparation of 1,8-dioxodecahydroacridines

Entry	Solvent	Time (h)	Yield (%)^a^
1	CH_3_CN	4.5	72
2	DCM	3.0	80
3	DMF	3.0	90
4	Ethanol	1.0	96
5	Solvent free	4.5	60

^a^Yields of isolated products

To evaluate the scope and the generality of this new protocol for various aldehydes and amines under optimized conditions. A series of aromatic aldehydes bearing either electron-donating or electron-withdrawing substituents reacted successfully with dimedone and aromatic amines or ammonium acetate to afford a wide range of substituted 1,8-dioxodecahydroacridines products in high yields in a shorter reaction time and the results are summarized in Table 3. We explored further the electronic effect of various substituents present on the aldehyde component. We noticed that a wide range of aldehydes having both electron-donating and electron-withdrawing substituents are equally facile for the reaction, and gave the corresponding 1,8-dioxodecahydroacridine derivatives in very good yields. We have observed most of the electron-donating aldehydes reacted in a shorter reaction time and gave the corresponding 1,8-dioxodecahydroacridine derivatives in good yields than electron-withdrawing aldehydes.

**Table 3:** Synthesis of 1,8-dioxodecahydroacridines catalyzed by cobalt–alanine metal complex under aqueous ethanol medium

Entry	Aldehyde (R)	R^1^ or NH_4_OAC	Product	Time (h)	Yield (%)^a^	Mp. (°C)
1	H	Ph	**4a**	1.0	96	252–254
2	4-OCH_3_	Ph	**4b**	1.5	90	220–222
3	4-CH_3_	Ph	**4c**	2.0	90	259–262
4	4-CN	Ph	**4d**	2.0	80	267–269
5	4-NO_2_	Ph	**4e**	2.5	85	265–267
6	4-OH	Ph	**4f**	2.0	88	232–233
7	4-Cl	Ph	**4g**	2.0	80	244–246
8	4-Br	Ph	**4h**	2.0	85	251–253
9	2-Cl	Ph	**4i**	2.5	75	249–252
10	2-NO_2_	Ph	**4j**	3.0	80	272–275
11	3-NO_2_	Ph	**4k**	3.0	80	293–295
12	4-OCH_3_	NH_4_OAC	**4l**	2.0	85	272–275
13	4-CH_3_	NH_4_OAC	**4m**	2.0	85	268–271
14	4-CN	NH_4_OAC	**4n**	2.5	80	281–283
15	4-NO_2_	NH_4_OAC	**4o**	3.0	80	285–287
16	2,3-(Cl)_2_	Ph	**4p**	3.0	85	273–276
17	2,3-(OCH_3_)_2_	Ph	**4q**	2.5	95	288–291
18	3,4-(NO_2_)_2_	Ph	**4r**	3.5	80	278–281
19	3,4-(NO_2_)_2_	NH_4_OAC	**4s**	4.0	75	281–283
20	2,3-(Cl)_2_	NH_4_OAC	**4t**	3.5	80	322–325
21	2,3-(OCH_3_)_2_	NH_4_OAC	**4u**	3.0	90	303–305
22	2-OH	Ph	**4v**	3.0	85	246–249

^a^Yields of isolated products

The physical and spectral data of synthesized compounds were (**4a**–**4v**) found to be in agreement with the reported data.

**Scheme 2:**
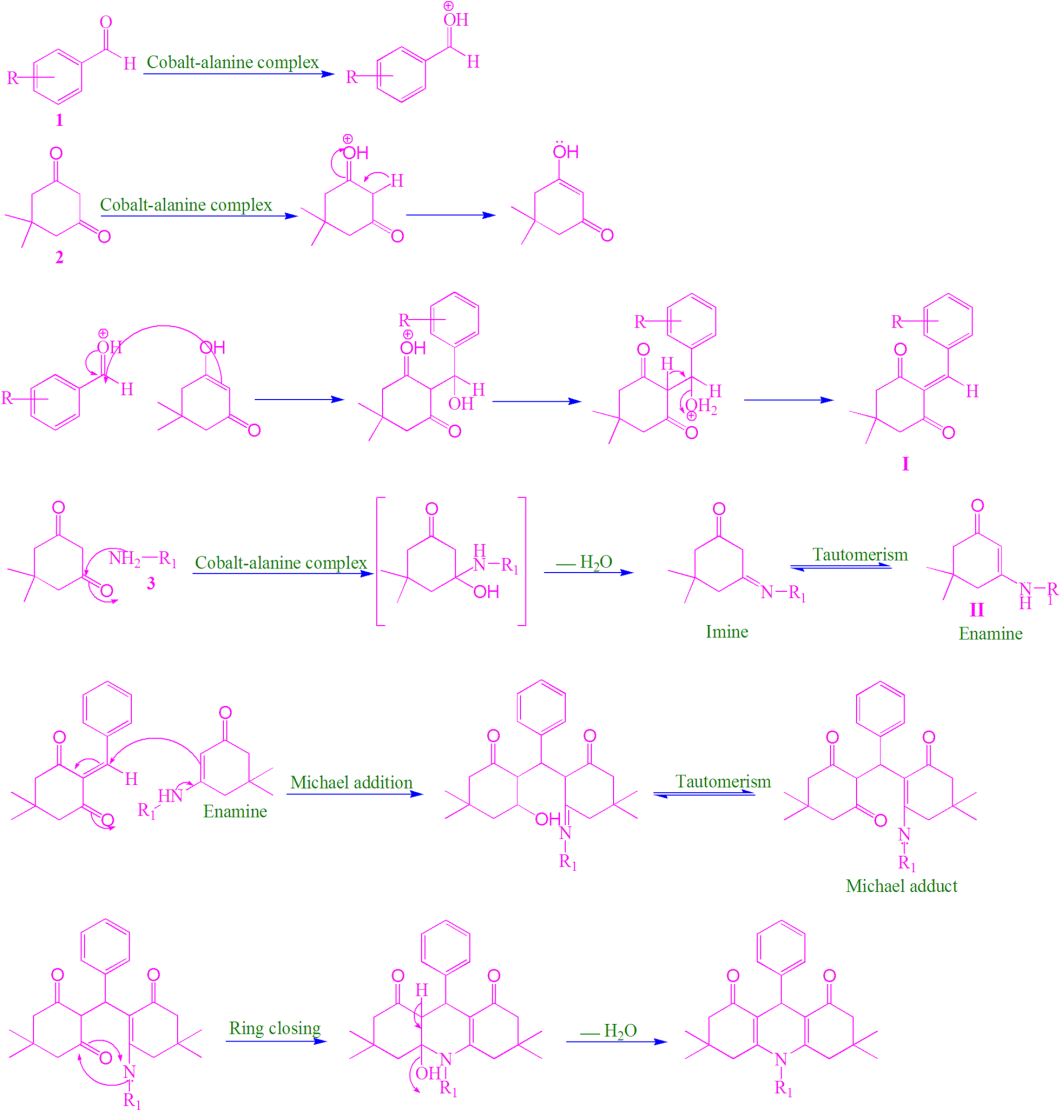
Plausible mechanism for the formation of the 1,8-dioxodecahydroacridines catalyzed by cobalt–alanine metal complex

A plausible mechanism for the formation of the 1,8-dioxodecahydroacridine products using cobalt–alanine metal complex as a catalyst has presented in Scheme 2.

We propose that the cobalt–alanine metal complex induces the polarization of the carbonyl groups, of aldehyde (1) and 1,3-diketone (2). Further, we assume that cobalt–alanine metal complex triggers the 1,3-diketone (2) and facilitates the formation of corresponding imine through a condensation reaction with amine 3. The obtained imine, further undergoes tautomerizes to form enamine. Therefore, cobalt–alanine metal complex facilitates the formation of two reactive intermediate species **I** and **II** which subsequently react each other via Michael addition and forms the adduct, which further tautomerize. The tautomerized Michael adduct undergoes simple intramolecular ring closure and give the final desired products **4a**–**4v** by loss of water molecule.

### Spectral data for selected compounds

#### 3,3,6,6-Tetramethyl-9,10-diphenyl-3,4,6,7-tetrahydroacridine-1,8(2H,5H,9H,10H)-dione (**4a**)

IR (KBr disc) cm^−1^: 3120, 2984, 2848, 1664, 1583, 1467, 1437, 1385, 1312, 1244, 1184, 1065, 860, 787, 714, 684. ^1^H NMR (500 MHz CDCl_3_): δ 0.86 (s, 6H, 2CH_3_), 0.94 (s, 6H, 2CH_3_), 2.14 (d, 2H, J = 16.4 Hz,), 2.23 (d, 2H, J = 16.4 Hz,), 2.24–2.41 (m, 4H, 2CH_2_), 5.52 (s, 1H), 7.01 (s, 1H, ArH), 7.04 (d, J = 6.2 Hz, 2H, ArH), 7.12–7.28 (m, 5H, ArH), 7.34 (d, J = 6.2 Hz, 2H, ArH). ^13^C NMR (125 MHz, CDCl_3_): δ 22.8, 23.4, 36.2, 42.2, 52.4, 123.8, 124.8, 126.6, 127.2, 128.2, 130.6, 132.2, 136.2, 145.8, 151.6, 212.8.

#### 3,3,6,6-Tetramethyl-9-(4-methoxyphenyl)-10-phenyl-3,4,6,7-tetrahydroacridine-1,8(2H,5H,9H,10H)-dione (**4b**)

IR (KBr disc) cm^−1^: 3118, 2964, 2902, 2882, 1654, 1614, 1604, 1572, 1502, 1484, 1342, 1274, 1228, 1168, 1058, 856, 734, 658. ^1^H NMR (500 MHz, CDCl_3_): 0.83 (s, 6H, 2CH_3_), 1.02 (s, 6H, 2CH_3_), 1.24 (d, 2H, J = 14.6 Hz,), 2.14 (d, 2H, J = 14.6 Hz,), 2.25–244 (m, 4H, 2CH_2_), 3.94 (s, 3H, OCH_3_), 5.29 (s, 1H), 6.64 (s, 1H, ArH), 6.98 (d, J = 7.6 Hz, 2H, ArH), 7.14–7.28 (m, 3H, ArH), 7.39 (d, J = 7.4 Hz, 2H, ArH). ^13^C NMR (125 MHz, CDCl_3_): δ 22.3, 23.8, 35.2, 42.4, 52.5, 125.2, 126.8, 127.5, 129.2, 131.5, 134.2, 136.9, 146.8, 148.3, 150.3, 166.2, 212.6.

#### 3,3,6,6-Tetramethyl-9-(4-cyanophenyl)-10-phenyl-3,4,6,7-tetrahydroacridine-1,8(2H,5H,9H,10H)-dione (**4d**)

IR (KBr disc) cm^−1^: 2984, 2912, 2284, 1672, 1548, 1484, 1322, 1277, 1231, 1164, 1123, 1112, 1052, 842, 722, 580. ^1^H–NMR (500 MHz, CDCl_3_): δ 0.72 (s, 6H, 2CH_3_), 0.92 (s, 6H, 2CH_3_), 1.62 (d, J = 14.5 Hz, 2H, CH_2_), 2.06 (d, J = 14.5 Hz, 2H, CH_2_), 2.11 (d, J = 12.2 Hz, 2H, CH_2_), 2.24 (d, J = 12.2 Hz, 2H, CH_2_), 5.28 (s, 1H), 7.22 (d, J = 6.0 Hz, 2H, ArH), 7.48 (d, J = 6.4 Hz, 2H, ArH), 7.52 (d, J = 6.0 Hz, 2H, ArH), 7.64 (m, 3H, ArH). ^13^C-NMR (125 MHz, CDCl_3_): δ 24.2, 29.8, 30.8, 32.2, 42.4, 52.4, 111.2, 113.8, 121.4, 127.6, 130.7, 132.7, 137.6, 150.2, 154.1, 195.2.

#### 3,3,6,6-Tetramethyl-9-(4-nitrophenyl)-10-phenyl-3,4,6,7-tetrahydroacridine-1,8(2H,5H,9H,10H)-dione (**4e**)

IR (KBr disc) cm^−1^: 2982, 1654, 1568, 1544, 1368, 1258, 1146, 1122, 1102, 1068, 882, 856, 724, 588, 526. ^1^H-NMR (500 MHz, CDCl_3_): δ 0.74 (s, 6H, 2CH_3_), 0.92 (s, 6H, 2CH_3_), 1.35 (d, J = 14.8 Hz, 2H, CH_2_), 2.08 (d, J = 14.8 Hz, 2H, CH_2_), 2.12 (d, J = 12.4 Hz, 2H, CH_2_), 2.18 (d, J = 12.4 Hz, 2H, CH_2_), 5.16 (s, 1H, CH), 7.14 (d, J = 7.2 Hz, 2H, ArH), 7.58 (d, J = 7.2 Hz, 2H, ArH), 7.62 (s, 1H, ArH), 7.68 (d, J = 8.2 Hz, 2H, ArH), 8.16 (d, J = 8.2 Hz, 2H, ArH). ^13^C-NMR (125 MHz, CDCl_3_): δ 24.2, 28.2, 31.6, 36.2, 42.4, 52.3, 113.2, 123.8, 129.2, 130.2, 136.4, 147.8, 151.4, 155.8, 195.8.

#### 3,3,6,6-Tetramethyl-9-(4-hydroxyphenyl)-10-phenyl-3,4,6,7-tetrahydroacridine-1,8(2H,5H,9H,10H)-dione (**4f**)

IR (KBr disc) cm^−1^: 3418, 2976, 2912, 2882, 1658, 1584, 1512, 1488, 1433, 1367, 1242, 1184, 1054, 822, 728, 712, 686. ^1^H NMR (500 MHz, CDCl_3_): δ 0.82 (s, 6H, 2CH_3_), 0.92 (s, 6H, 2CH_3_), 1.48 (d, J = 15.4 Hz, 2H), 2.02 (d, J = 15.4, 2H), 2.28–248 (m, 4H, 2CH_2_), 5.48 (s, 1H), 7.12 (d, J = 6.8 Hz, 2H, ArH), 7.18–7.23 (m, 4H, ArH), 7.36 (d, J = 6.4 Hz 2H, ArH), 9.11 (s, 1H, OH). ^13^C NMR (125 MHz, CDCl_3_): δ 22.8, 23.6, 33.2, 41.2, 55.2, 123.2, 127.8, 129.2, 131.9, 136.2, 138.9, 148.4, 152.7, 153.5, 165.2, 212.6.

#### 3,3,6,6-Tetramethyl-9-(4-chlorophenyl)-10-phenyl-3,4,6,7-tetrahydroacridine-1,8(2H,5H,9H,10H)-dione (**4g**)

^1^H-NMR (500 MHz, CDCl_3_): δ 0.78 (s, 6H, 2CH_3_), 0.82 (s, 6H, 2CH_3_), 1.69 (d, J = 16.6 Hz, 2H, CH_2_), 2. 21 (d, J = 16.6 Hz, 2H, CH_2_), 2.08 (d, J = 14.8 Hz, 2H, CH_2_), 2.14 (d, J = 14.8 Hz, 2H, CH_2_), 5.21 (s, 1H, CH), 7.14 (d, J = 7.6 Hz, 2H, ArH), 7.21 (d, J = 7.6 Hz, 2H, ArH), 7.38 (d, J = 8.6 Hz, 2H, ArH), 7.44 (m, 3H, ArH).

#### 3,3,6,6-Tetramethyl-9-(4-bromophenyl)-10-phenyl-3,4,6,7-tetrahydroacridine-1,8(2H,5H,9H,10H)-dione (**4h**)

IR (KBr disc) cm^−1^: 3024, 2986, 2848, 1665, 1642, 1564, 1474, 1432, 1422, 1402, 1384, 1342, 1248, 1218, 1184, 1136, 1054, 1038, 1024, 936, 875, 741, 712, 684. ^1^H NMR (500 MHz, CDCl_3_): δ 0.74 (s, 6H, 2CH_3_), 0.86 (s, 6H, 2CH_3_), 1.72 (d, J = 16.8 Hz, 2H, 2CH), 2.18 (d, J = 16.8 Hz, 2H, 2CH), 2.21–2.28 (m, 4H, 2CH_2_), 5.18 (s, 1 H, CH), 7.26 (d, J = 6.8 Hz, 2H, ArH), 7.42–7.48 (m, 4H, ArH), 7.62–7.66 (m, 3H, ArH). ^13^C NMR (125 MHz, CDCl_3_): δ 24.6, 28.2, 31.8, 33.6, 43.5, 51.2, 113.2, 117.7, 128.2, 129.8, 130.6, 132.6, 139.1, 145.2, 151.2, 196.8.

#### 3,3,6,6-Tetramethyl-9-(3-nitrophenyl)-10-phenyl-3,4,6,7-tetrahydroacridine-1,8(2H,5H,9H,10H)-dione (**4k**)

^1^H-NMR (500 MHz, CDCl_3_): δ 0.82 (s, 6H, 2CH_3_), 0.94 (s, 6H, 2CH_3_), 1.78 (d, J = 16.6 Hz, 2H, CH_2_), 2.08 (d, J = 16.4 Hz, 2H, CH_2_), 2.16 (d, J = 13.6 Hz, 2H, CH_2_), 2.28 (d, J = 13.6 Hz, 2H, CH_2_), 5.42 (s, 1H, CH), 7.34 (m, 1H, ArH), 7.46 (m, 2H, ArH), 7.64 (m, 3H, ArH), 7.94 (m, 2H, ArH), 8.38 (s, 1H, ArH).

#### 3,3,6,6-Tetramethyl-9-(4-methoxyphenyl)-3,4,6,7-tetrahydroacridine-1,8(2H,5H,9H,10H)-dione (**4l**)

IR (KBr disc) cm^−1^: 3184, 1648, 1617, 1462, 1366, 1223, 1138, 1084, 968, 748, 646. ^1^H NMR (500 MHz, CDCl_3_): δ 0.94 (s, 6H, 2 CH_3_), 1.05 (s, 6H, 2CH_3_), 2.09–2.16 (dd, 4H), 2.19–2.28 (m, 4H, 2 CH_2_), 3.66 (s, 3H), 5.04 (s, 1H), 6.69–6.73 (d, J = 8.4 Hz, 2H, ArH), 7.23–7.26 (d, J = 7.6 Hz, 2H, ArH), 8.22 (s, 1H, NH).

#### 3,3,6,6-Tetramethyl-9-(4-nitrophenyl)-3,4,6,7-tetrahydroacridine-1,8(2H,5H,9H,10H)-dione (**4o**)

^1^H-NMR (500 MHz, CDCl_3_) δ 0.98 (s, 6H, 2CH_3_), 1.08 (s, 6H, 2CH_3_), 2.14 (d, J = 16.5 Hz, 2H), 2.34 (d, J = 16.2 Hz, 2H), 2.38 (d, J = 14.8 Hz, 2H), 2.52 (d, J = 14.8 Hz, 2H), 5.24 (s, 1H, CH), 6.18 (s br., 1H, NH), 7.68 (d, J = 7.8 Hz, 2H, arom-H), 8.16 (d, J = 7.8 Hz, 2H, arom-H).

#### 3,3,6,6-Tetramethyl-9-(2,3-dichlorophenyl)-10-phenyl-3,4,6,7-tetrahydroacridine-1,8(2H,5H,9H,10H)-dione (**4p**)

IR (KBr disc) cm^−1^: 2954, 2936, 1658, 1642, 1585, 1464, 1342, 1268, 1176, 1049, 1024, 842, 738, 582. ^1^H-NMR (500 MHz, CDCl_3_): δ 0.84 (s, 6H, 2CH_3_), 0.98 (s, 6H, 2CH_3_), 1.76 (d, J = 17.6 Hz, 2H, CH_2_), 2.16 (d, J = 17.6 Hz, 2H, CH_2_), 2.34 (m, 4H, CH_2_), 5.28 (s, 1H, CH), 7.24 (m, 2H, ArH), 7.38 (m, 2H, ArH), 7.48 (s 1H, ArH), 7.52 (m, 3H, ArH). ^13^C-NMR (125 MHz, CDCl_3_): δ 26.2, 30.4, 31.6, 43.2, 51.8, 113.2, 126.8, 129.2, 130.2, 131.3, 133.2, 136.5, 145.2, 151.7, 196.2.

#### 3,3,6,6-Tetramethyl-9-(2,3-dimethoxyphenyl)-10-phenyl-3,4,6,7-tetrahydroacridine-1,8(2H,5H,9H,10H)-dione (**4q**)

IR (KBr disc) cm^−1^: 3412, 1654, 1454, 1382, 1231, 1130, 1065, 978, 768, 645. ^1^H NMR (500 MHz, CDCl_3_): δ 0.88 (s, 6H, 2CH_3_), 0.94 (s, 6H, 2CH_3_), 1.86–2.24 (dd, 4H), 2.32–2.46 (dd, 4H), 3.84 (s, 3H, OCH_3_), 3.94 (s, 3H, OCH_3_), 5.12 (s, 1H), 6.72–6.84 (m, 3H, ArH), 9.12 (s, 1H, NH).

## Conclusion

In conclusion, we have developed a facile and an efficient protocol for the synthesis of 1,8-dioxodecahydroacridines using cobalt–alanine metal complex as a catalyst in aqueous ethanol as solvent via one-pot three-component condensation of aromatic aldehydes, dimedone, aniline or ammonium acetate. Significant advantages of this study are reasonably simple experimental workup procedure and catalyst preparation, ease of product isolation, high to excellent yields, short reaction time and using catalytic amount of cobalt–alanine metal complex, are notable advantages of the present methodology.

## Experimental procedure

### Materials and methods

All chemicals were purchased from Sigma Aldrich and used as received without further purification. All chemicals and reagents used in the present study were of analytical grade. The reactions were monitored by TLC using silicagel plates. The FTIR spectra were recorded on a Shimadzu JASCO FTIR-460 plus spectrometer using KBr pellets or neat. The UV–visible spectra of the compounds were recorded on Shimadzu UV-2100 spectrophotometer. The morphology of synthesized complex was characterized by Scanning electron microscopy (SEM) on a JEOL-JSM-6390 LV. ^1^H NMR and ^13^C NMR spectra were recorded on a Brucker DRX 500 AVANCE (500 MHz) spectrometer using CDCl_3_ as solvent and TMS as internal standard. The elemental analysis of the complexes were recorded by using Perkin-Elmer CHN-2400 analyzer their results were found to be good agreement with the calculated values. Photoluminance spectra of the complexes and ligands were recorded on LUMINA fluorescence spectrometer of Thermo Scientific Co. USA. The XRD measurements were performed on Schimadzu DX-6000 using Cu for Kα-particle source.

#### General procedure for the preparation of cobalt–alanine complex

**Fig. 1:**
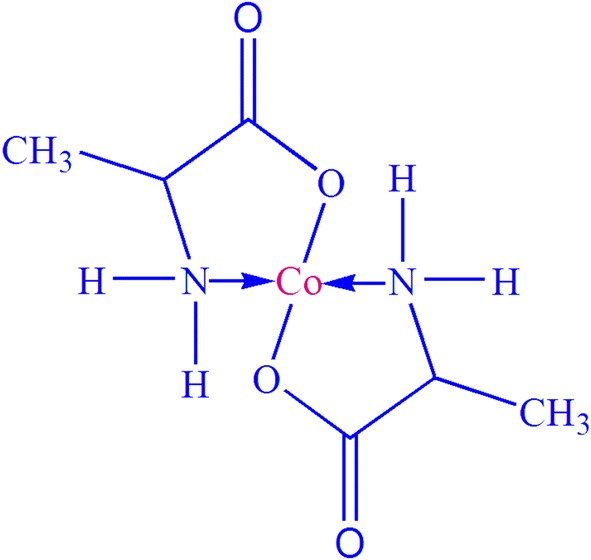
Probable structure for Co–alanine metal complex

It is commonly well-known that a metal ion can bind two amino acids to form an amino acid metal complex (Fig. 1). Therefore, amino acid-metal complexes were prepared in hot ethanol solvent by reacting the corresponding metal ion and amino acid in a 1:2 molar ratio.

**Scheme 3:**
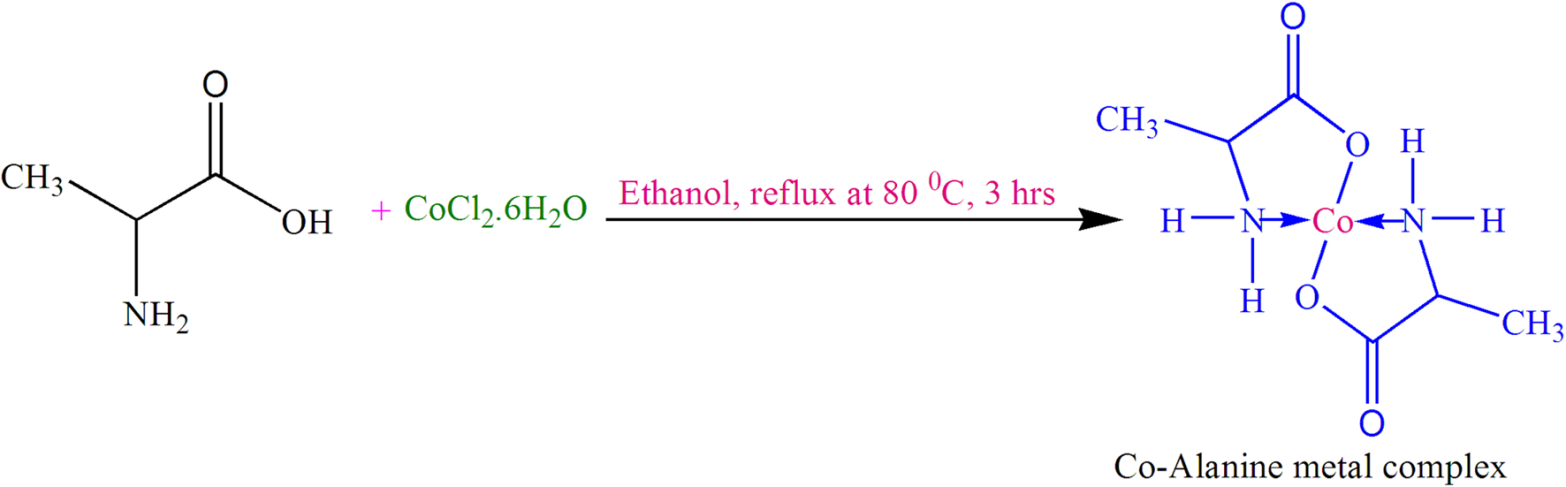
Synthesis of cobalt–alanine metal complex

The synthesis of cobalt–alanine metal complex was achieved in a two steps. In a first step, the CoCl_2_·6H_2_O (2.4 g) metal salt was dissolved in a hot ethanol solvent and l-alanine (1.8 g) amino acid ligand also dissolved in ethanol separately. In the second step, the resultant solution of ligand was slowly added drop by drop into the metal salt solution under vigorous stirring. Once the addition of ligand solution was completed, then 0.01 M Na_2_CO_3_ solution also added slowly to adjust the P^H^ around 6.5 to 8.5 for the formation of Co–alanine metal complex. The reaction mixture was refluxed for 3 h at 80 °C under vigorous stirring (Scheme 3) [56–58]. The reaction progress was observed by TLC. The mobile phase condition was *n*-butanol:acetone:acetic acid:water (7:7:2:4) used as a solvent system. The TLC plate was run in this solvent system and the obtained amino acid metal complex was moved well on the TLC plate and also visualized by using solution of ninhydrin. After completion of the reaction, as indicated by TLC, the mixture was allowed to cooled at room temperature; thus the obtained solid crude product was separated by filtration and the crude product was recrystallized in acetone and diethyl ether solvent system with constant stirring under reflux for a period of time, later it was allowed to cool. The resultant solid product was filtered and dried under vacuum. The obtained pure solid product was in a purple color.

IR (KBr, ν cm^−1^): 3084 (ν_OH_), 1620 (ν_C=N_), 1014 (ν_Co–O_), 485 (ν_Co–N_).

Elemental analysis: C: 30.14%, H: 5.52%, N: 11.82%, Co: 25.24%.

**Fig. 2:**
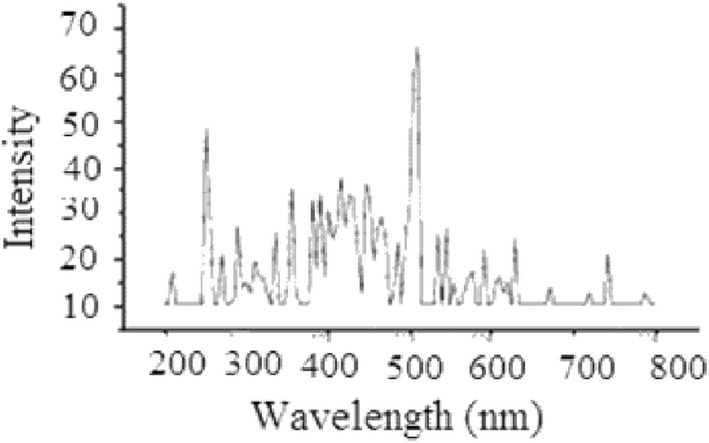
Photoluminence (PL) emission spectra for Co–alanine metal complex

The synthesized cobalt–alanine metal complex was characterized by using photoluminescence spectroscopy, which provided an evidence of complexation. The recorded emission spectra showed an interesting evidence for the complex formation. The emissions at λ_max_ 556–566 and 660–730 nm provide evidence that the metal atoms are transferring energy to the ligand (alanine) hence; promoting the photoluminescence to the organic ligand (Fig. 2).

**Fig. 3:**
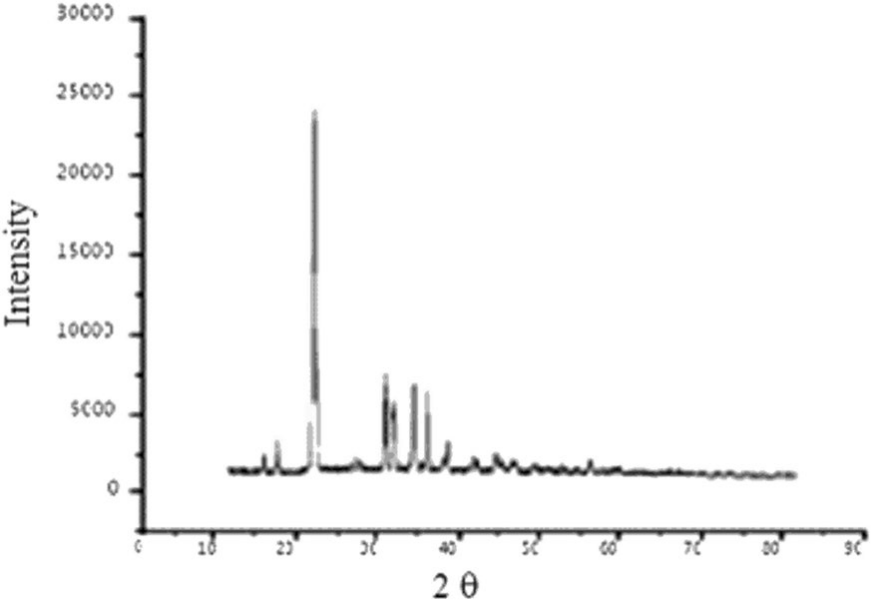
The XRD of cobalt–alanine metal complex

Further evidence to suggest this justification was revealed by the powder XRD analysis of the complex. The obtained new peaks were observed at 30–40 θ which clearly provides the evidence for the formation of complex (Fig. 3).

**Fig. 4:**
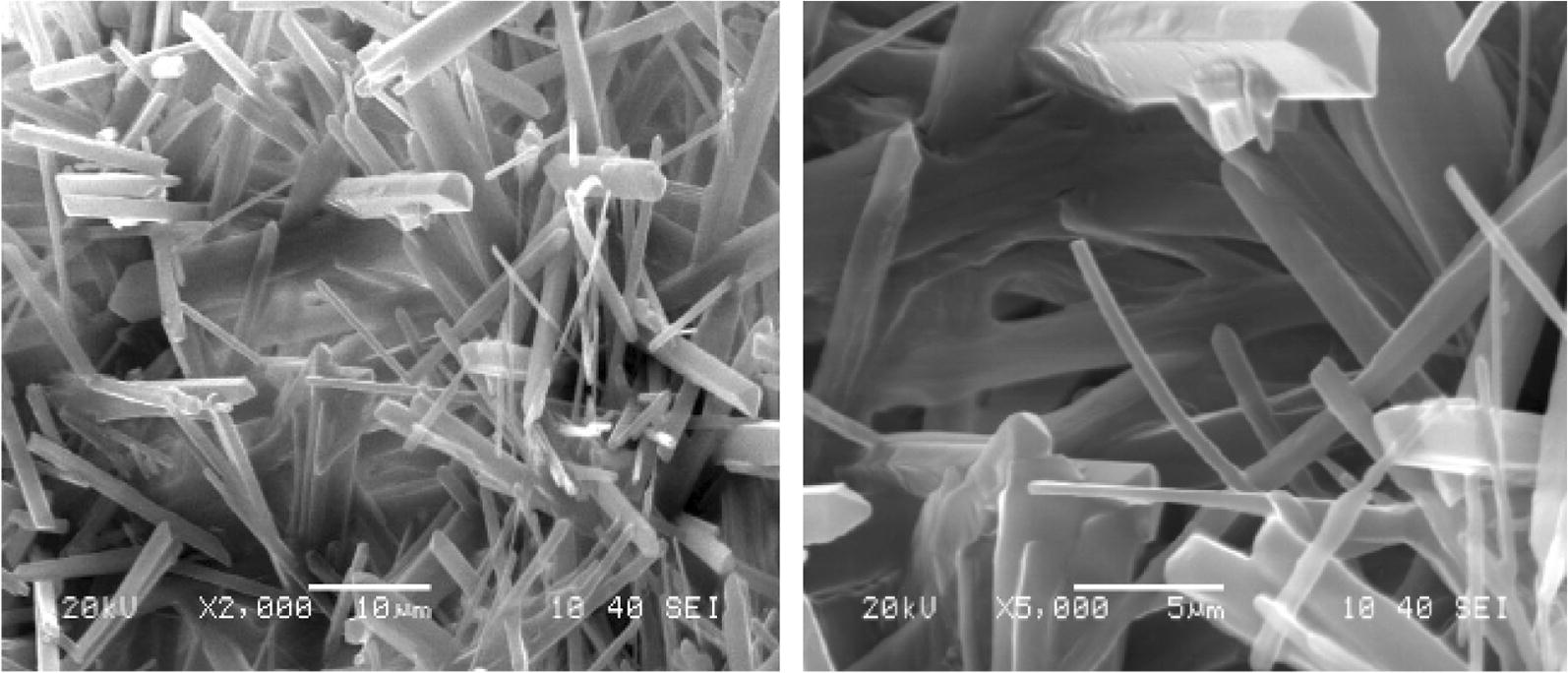
SEM micrograph of Co–alanine complex; at different magnifications showing the structure of rods

The final evidence of complex composition was predicted from by SEM analysis. The SEM-micrograph for the cobalt–alanine has shown in Fig. 4. The morphology, texture and shape of the synthesized complex with varying thickness in the range of 5 to 10 µm are seen. The morphology of this complex was seen as rods at different magnifications.

#### General procedure for the synthesis of 1,8-dioxodecahydroacridines catalyzed by cobalt–alanine metal complex

**Scheme 4:**
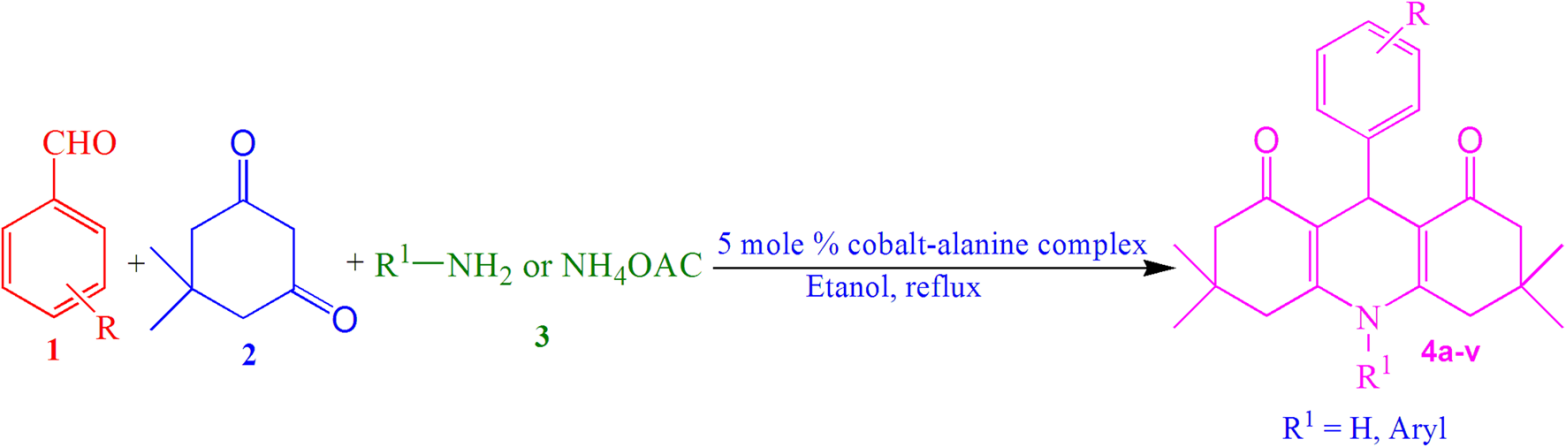
Synthesis of 1,8-dioxodecahydroacridines **4a**–**v** catalyzed by cobalt–alanine complex

A mixture of an aromatic aldehyde **1** (1 mmol), 5,5-dimethyl-1,3-cyclohexanedione **2** (2 mmol), aromatic amine or ammonium acetate **3** (1.2 mmol) and cobalt–alanine complex (5 mol%) in ethanol (10 mL) was stirred at reflux. The progress of the reaction was monitored by TLC. After completion of the reaction as indicated by TLC, the mixture was cooled to room temperature and filtered. The filtrate was concentrated to obtained the crude product. The crude products were purified and recrystallized from EtOH and water mixture to obtain pure products in high yields (Scheme 4).

## Abbreviations

MCR: multi-component reactions
DHPs: 1,4-dihydropyridines
SBSSA: sulfonic acid functionalized silica
TEBAC: triethylbenzylammoniumchloride
SBNPSA: silica-bonded *N*-propyl sulfamic acid
[Hmim]TFA: 1-methyl imidazolium trifluoroacetate
DBSA: *p*-dodecylbenzenesulfonicacid
DCM: dichloromethane
DMF: dimethylformamide
EtOH: ethanol
SEM: scanning electron microscope
FT-IR: Fourier transform infrared spectroscopy
TLC: thin layer chromatography
